# *Euphorbia* Section *Hainanensis* (Euphorbiaceae), a New Section Endemic to the Hainan Island of China From Biogeographical, Karyological, and Phenotypical Evidence

**DOI:** 10.3389/fpls.2018.00660

**Published:** 2018-05-18

**Authors:** Xinmin Tian, Qiuyan Wang, Yongfeng Zhou

**Affiliations:** ^1^Department of Biological Sciences, College of Life Science and Technology, Xinjiang University, Urumqi, China; ^2^Key Laboratory of Oasis Ecology, Ministry of Education, Institute of Arid Ecology and Environment, Xinjiang University, Urumqi, China; ^3^Department of Ecology and Evolutionary Biology, University of California, Irvine, Irvine, CA, United States; ^4^School of Life Science, Lanzhou University, Lanzhou, China

**Keywords:** phylogeny, biogeography, endangered species, limestone mountains, long distance dispersal

## Abstract

*Euphorbia hainanensis* is an endangered species endemic to the tropical Hainan Island in southern China and of historical importance for Chinese medicine. It is currently the only unplaced species of the genus *Euphorbia* (Euphorbiaceae) due to its isolated island distribution and debated placement by a previous molecular phylogenetic study. We sequenced nuclear ITS and chloroplast *rbcL* and *ndhF* for newly collected accessions of *E. hainanensis* and additional *Euphorbia* species found in Hainan, and analyzed the sequences in the context of the entire genus together with published data. All gene regions highly supported that *E. hainanensis* occupied an isolated phylogenetic position, showing no close affinity with any known *Euphorbia* sections suggesting it was a new section. ITS placed *E. hainanensis* sister to sect. *Crossadenia* (subgenus *Chamaesyce*) from Brazil with an estimated divergence time of 9.3-30.6 Mya while the chloroplast markers placed *E. hainanensis* at a position sister to the entire New World clade of *Euphorbia* subgenus *Chamaesyce*. In addition, our karyological results suggested a close affinity between *E. hainanensis* and the New World species of *Euphorbia* subg. *Chamaesyce*, with which shared the same chromosome number 2n = 28 and basic chromosome number x = 7. Phenotypically, *E. hainanensis* is unique with no close resemblance to other species in *Euphorbia* subg. *Chamaesyce*. Based on its isolated biogeographical, karyological, and phenotypical position, we propose a new section *E*. subgenus *Chamaesyce* section *Hainanensis* that might origin from long distance dispersal events because collective evidences showed a close affinity between the species from the Old World with those from the New World.

## Introduction

*Euphorbia* L. (Euphorbiaceae) is one of the largest genera of seed plants with ~2,000 species worldwide and is especially diverse in the tropics and subtropics (Horn et al., 2012). During the past 20 years, molecular phylogenetic studies have made much progress in understanding the broad scale relationships for *Euphorbia*, which have discovered four subgenera: *E*. subg. *Rhizanthium, E*. subg. *Esula, E*. subg. *Euphorbia*, and *E*. subg. *Chamaesyce*, respectively (Steinmann and Porter, 2002; Bruyns et al., 2006, 2011; Park and Jansen, 2007; Thakur and Patil, 2011; Yang and Berry, 2011; Horn et al., 2012; Yang et al., 2012; Dorsey et al., 2013; Peirson et al., 2013; Riina et al., 2013). Subgenus *Chamaesyce* mainly occurs in the New world while the rest three subgenera are mostly distributed in the Old World (Yang et al., 2012; Peirson et al., 2013). Molecular phylogenetic studies had also suggested that the evolution of some characters in *Euphorbia*, such as growth form and cyathial form are highly homoplasious, and this genus had experienced frequent long-distance dispersal events that led to its worldwide distribution (Steinmann and Porter, 2002; Haevermans et al., 2004; Bruyns et al., 2006, 2011; Park and Jansen, 2007; Yang and Berry, 2011; Horn et al., 2012; Yang et al., 2012; Dorsey et al., 2013; Peirson et al., 2013; Riina et al., 2013).

**Figure 1:**
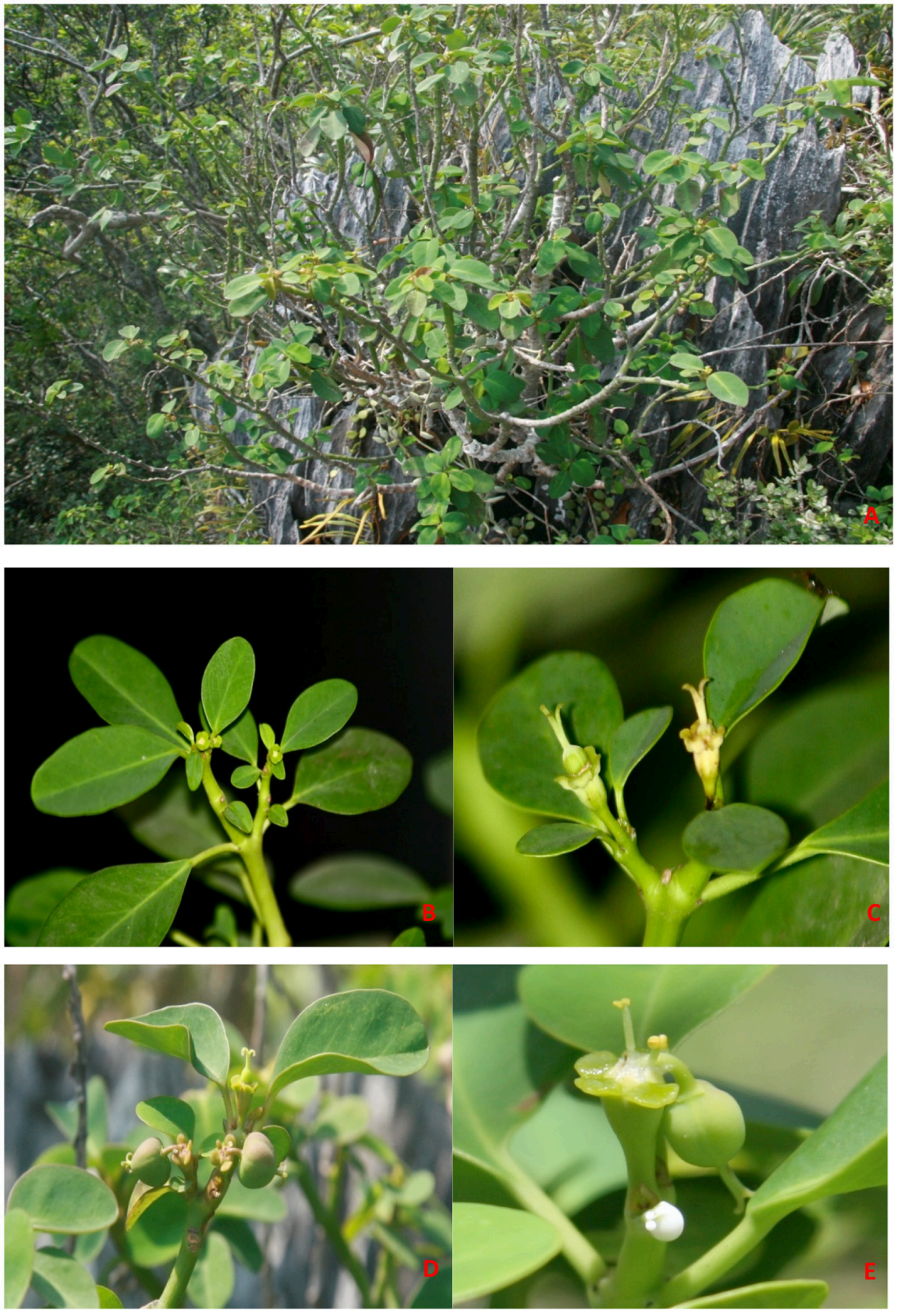
*Euphorbia hainanensis*. **(A)** Habit on the limestone. **(B–E)** Detail of flower and fruit. **(B)** Terminal solitary cyathium. **(C)** Cyathia with involucral glands without appendages. **(D–E)** Cyathium with young fruits.

*Euphorbia hainanensis* Croizat is an endangered woody *Euphorbia* species endemic to the Hainan Island in southern China (Li, 1994; Wu, 2007; Zhang et al., 2007). It occurs in three fragmented natural populations on top of limestone mountains in the Exianling protected region in the Hainan Island (Figure 1). It has been considered as important medical resources because of the diterpenoid compound within this genus (Wu, 2007; Shi et al., 2008; Zhang et al., 2010; Ernst et al., 2015). Due to its remote distribution and difficulties in accessing the plant materials, so far, no studies have focused on its phenotype, phylogeny, and karyology. *Euphorbia hainanensis* has been postulated to be associated to *E*. subgenus *Poinsettia* (Li, 1994; Wu, 2007). Subgenus *Poinsettia* shared common characters including alternate leaves, very small caduceus stipules, cyathia in congested terminal cymes, unequal cyathophylls, and a single cyathial glands without appendages. Of the five species of this subgenus, only *E. hainanensis* is endemic in China and other four species are introduced from North America. Others suggested that *E. hainanensis* may be closely related to *E. dentata* because of their shared involucre glands without appendages (Niu, 2011). However, *E. dentata* is a naturalized species, which origins from the New World, and it is unlikely to be relatives of *E. hainanessis* in Hainan. Previous studies did not include this species because lack of materials, except phylogenetic analyses for subgenus *Chamaesyce* based on internal transcribed spacers (ITS) sequence that proposed weak supports for a lineage sect. *Crossadenia* comprising *E. hainanensis*, and other species mainly from the New World (Yang et al., 2012). The chromosomal structure from karyological analyses are very informative in systematic and evolutionary studies (Stebbing, 1971; Raven, 1975). Up to now, the chromosomal characters of *E. hainanensis* remain unknown.

In this study, we sequenced nuclear ITS and plastid *ndhF* and *rbcL* sequences from newly collected *E. hainanensis* accessions and other *Euphorbia* species that occur in Hainan in the context of the entire genus including published data to investigate the phylogenetic placement of *E. hainanensis*. Together with karyological and phenotypical analyses, we discuss the evolutionary and biogeographic origin of this enigmatic species.

## Materials and methods

### Taxon sampling

We sampled two accessions of *E. hainanensis* and one accession for each of seven additional *Euphorbia* species that occur on the Hainan Island in our phylogenetic analyses (*E. atoto, E. heterophylla, E. cyathophora, E. hirta, E. thymifolia, E. serrulata*, and *E. hyssopifolia*). Phenotypes including leaf, flower, and fruits were observed in natural populations and vouchers for all field-collected materials were deposited at Xinjiang University. Silica-preserved leaf materials were collected from the field collections for DNA analyses. In addition, published sequences from 83 *Euphorbia* species that covered all four subgenera and most sections (including all sections sister to sect. *Crossadenia*) were also included in phylogenetic analyses with *Euphorbia tithymaloides* (*Pedilanthus tithymaloides*) used as an outgroup. Species information and provenances of all species were listed in the Appendix A.

### DNA isolation and sequencing

Total genomic DNA was extracted using a High Performance (HP) Plant DNA Kit (TIANGEN BIOTECH) following the instructions. Primers were selected for this study based on previous phylogenetic studies of Euphorbiaceae (Appendix B). The PCR amplification was performed in a 25 μL PCR reaction system with about 20 ng DNA template, 12.5 μL of 2 × EcoTaq polymerase reaction Supermix, 1 μL of each primer (5 mol/μL), and sterile water. The sequence amplification program consisted of an initial template denaturation step at 94°C for 5 min, then 33 cycles of 94°C for 60 s, 55°C for 30 s, 72°C for90 s, and a final extension step of 72°C for 6 min. Finally, PCR products were purified with a CASpure PCR Purification Kit and sequenced by Sangon Biotech (Shanghai, China) using an ABI3730xL DNA Analyzer.

### Phylogenetic analyses

All sequences were aligned with software MEGA 6.06 (Tamura et al., 2013). Alignments were manually adjusted and refined with high quality sequences kept for the downstream analyses. Three datasets [ITS, cp DNA (*ndhF* and *rbcL*), and combined ITS and cp DNA] were used to do the phylogenetic analyses. We conducted an incongruence length difference (ILD) test to check the congruence between the ITS and cpDNA sequence. The ILD test was performed with 1000 replicates of the heuristic searches using TBR branch-swapping model in PAUP^*^ version 4.010b (Swofford, 2002). Gaps were considered as missing characters in all phylogenetic analyses. PAUP^*^ version 4.010b was used for conducting maximum parsimony (MP) and maximum likelihood (ML) analyses and constructing phylogenetic trees. To construct MP trees, we select MULTREES and TBR branch swapping as heuristic searches. Hundred repetitions of random sequence additions were used to calculate starting trees and the saved trees were obtained by stepwise addition. To evaluate support for nodes, bootstrap values (BP) were applied and calculated (Felsenstein, 1985). Software MODELTEST (Posada and Crandall, 1998) was employed to select the best-fit ML substitution model and ML trees were constructed using the simple addition. Branches support was computed by bootstrap analysis with 1,000 replicates (Guindon and Cascuel, 2003). Bayesian trees were reconstructed using MrBayes version 3.1.2 (Ronquist et al., 2012) and the same model of DNA evolution as for the ML analysis was selected. The Markov chain Monte Carlo (MCMC) algorithm was run over 2,000,000 generations to get the best convergence of the chain and one tree was saved every 100 generations. The first 20% trees were deleted as burn-in and the last 16,000 trees were assumed to calculate posterior possibilities (PP). Additionally, the Bayesian trees were viewed and saved by the software Figtree v1.4.3.

### The estimation of the divergence time

We could not collect any reliable fossil records that could be used for divergence time analysis, so we used ITS dataset to estimate the divergence time between section*. Hainanensis* and section *Crossadenia* with the BEAST software. Before the BEAST analysis, we used a likelihood test (LRT) to check if the strict clock model was suitable for our analysis (Huelsenbeck and Rannala, 1997). The results showed that molecular clock could not be rejected for the ITS data set and GTR + G model was set as the inference parameter. Then, BEAST version 1.8.0 (Drummond et al., 2012) was applied to calculate the genetic divergence based on a relaxed molecular clock tree. After a burn-in of 1000,000 steps, all data were collected once every 1000 steps from 40,000,000 MCMC steps. We tested convergence of the chains with the program Tracer v1.7 (Rambaut et al., 2018). Finally, we estimated the genetic divergence times between the section *Hainanensis* and section *Crossadenia* with a ITS substitution rates of 3.3 and 7.9 × 10^−9^ substitutions per site per year reported for most perennial herb or shrub genera (Richardson et al., 2001). We employed the software Figtree v1.4.3 to browse the constructed trees and divergence time.

### Karyological investigations

Seeds of *E. hainanensis* were collected from the natural population in Changjiang County, Hainan province (N 19° 00.779′, E 109° 06.836′, Alt. 1,018 m). They were germinated in petri dishes lined on gauze with moist condition. The root tips were pretreated in colchicines (0.1% w/v) for 2.5 h when they grow up to a length of 0.4-0.8 cm, then fixed in the Carnoy's fluid at 4°C for more than 1 h. The fixed shoots were hydrolysed in 1 mol/L HCl at 60°C for 8–12 min, and then washed with clean water, finally stained with carbol fuchsin dyes for about 1 min and squashed for microscope observation. The metaphase chromosomes of at least 15 cells of five seeds were investigated and counted.

## Results

**Figure 2:**
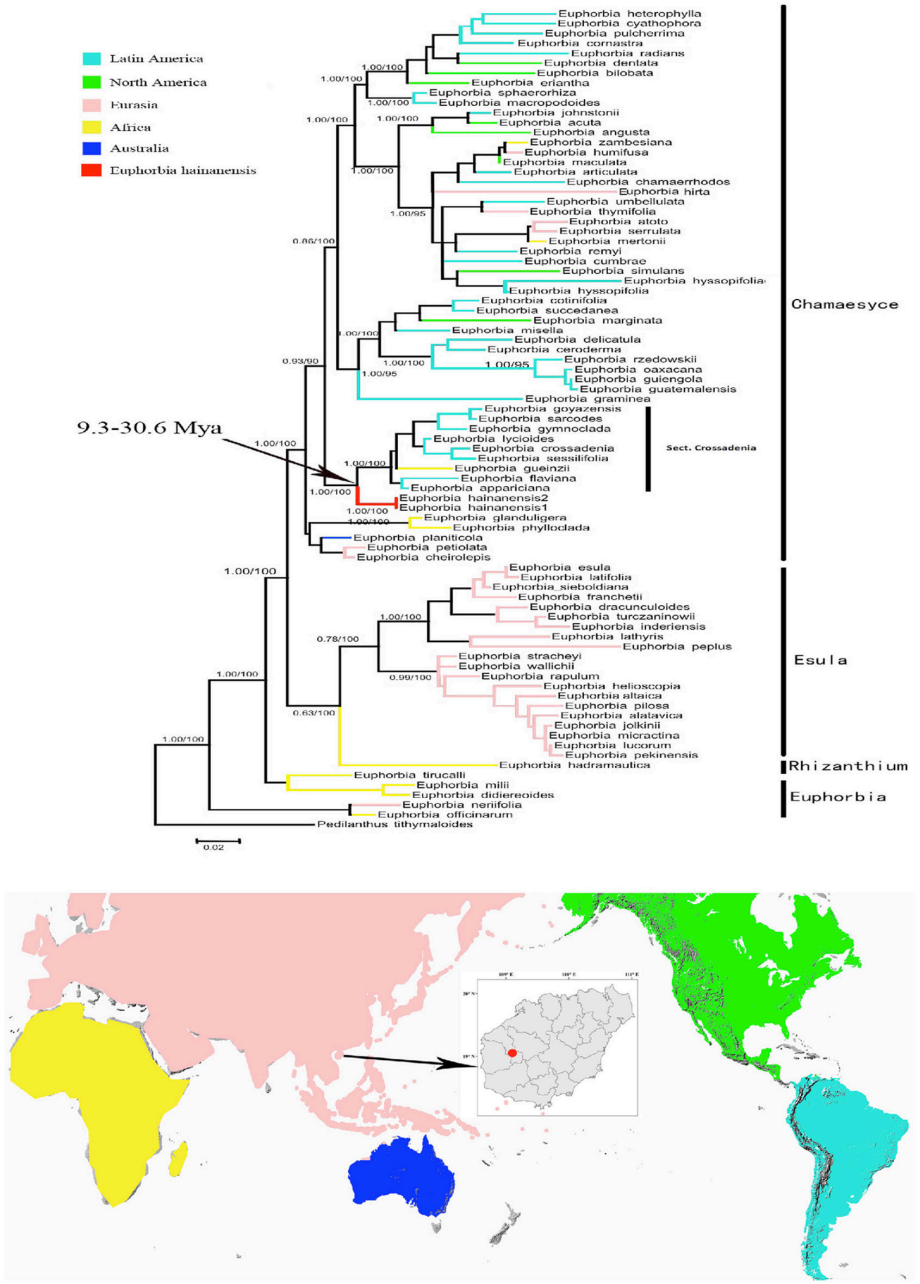
Biogeographic pattern of the *Euphorbia*. (Top) Maximum Likelihood tree based on analysis of the ITS data set. Numbers on branches were Bayes posterior probabilities/MP bootstrap values. Four subgenera groups were labeled as *Chamaesyce, Esula, Rhizanthium*, and *Euphorbia*, respectively. Branches leading to *E. hainanensis* were colored in red. (Bottom) Distribution of *Euphorbia* subgenera were showed in the map with continents colored the same on the phylogenic tree.

Our ITS matrix contained 83 accessions, of which eight were newly generated by our efforts. The aligned ITS dataset included 689 characters, of which 484 were variable (74 parsimony-uninformative and 410 parsimony-informative) while the other 195 characters were constant. In general, the phylogeny agreed with previous analyses (e.g., Yang et al., 2012; Peirson et al., 2013) with clear split of the four subgenera (Figure 2). The subge. *Chamaesyce* mainly occurred in the New World while the rest three subgenera were mostly distributed in the old world. The resulting placement for *E. hainanensis* were congruent among MP, ML and Bayesian analyses (Figure 2). *E. hainanensis* was highly supported to form a monophyletic clade with subg. *Chamaesyce* sect. *Crossadenia* from Brazil (PP = 1.00; BP = 100), with no close affinity with other species occurred in Hainan or the rest of the Old World (Figure 2).

Compared to ITS, the cpDNA matrix had a lower percentage of both variable and parsimony informative sites because of the low substitution rate for cpDNA. The concatenated *rbcL* and *ndhF* dataset consisted of 1,783 characters in total, with 114 variants but parsimony-uninformative, 138 potentially parsimony-informative and 1,531 invariable. The placement of *E. hainanensis* was congruent among the MP, ML and Bayesian analyses (Appendix 2). Instead of forming a monophyletic group with sect. *Crossadenia*, cpDNA highly supported *E. hainanensis* being sister to the entire New World clade *E*. subg. *Chamaesyce* (PP = 1.00; BP = 100). Given the much denser taxon representation of *ndhF* sequences in the GenBank than *rbcL*, we further constructed a *ndhF* matrix containing 56 species: consisted of 1,441 characters; 189 variants but parsimony-uninformative, 273 potentially parsimony-informative, and 979 invariable. Further *ndhF* phylogenetic analysis also recovered the same results as combined *rbcL* and *ndhF* dataset that *E. hainanensis* did not form a monophyletic group with sect. *Crossadenia*, but branched at the base of sect. *Tenellae* with high support (PP = 1.00, Appendix 1).

**Figure 3:**
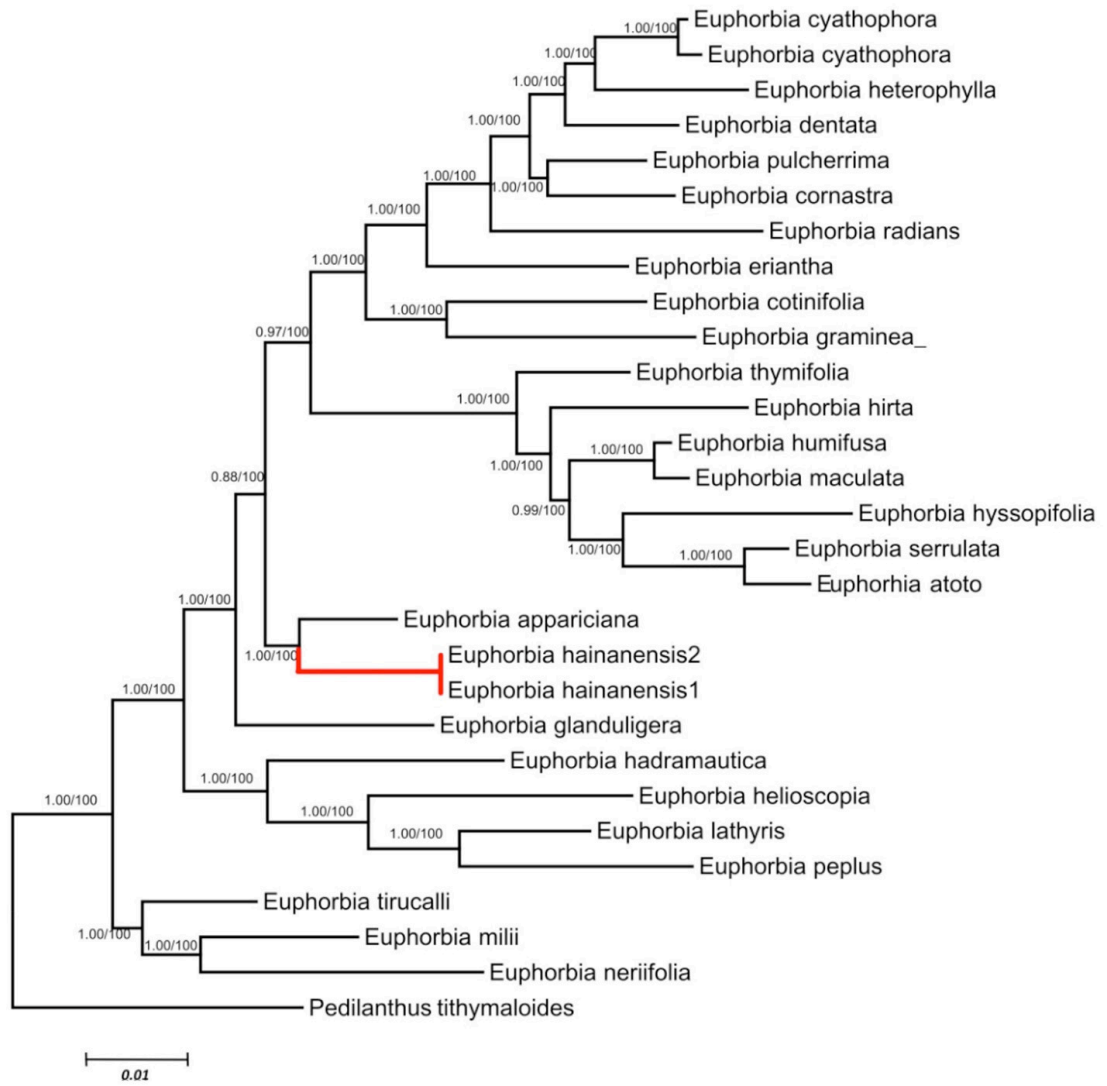
Maximum Likelihood tree based on combined ITS and cpDNA dataset. Numbers before slashes were Bayes posterior probabilities and the numbers after slashes indicated MP bootstrap values. The branches leading to *E. hainanensis* were marked in red.

We retained data for all *Euphorbia* species while both ITS and cp DNA sequences were obtainable. These two datasets are primarily congruent because the ILD test showed a significant difference (*P* > 0.01). Phylogenetic analyses generated almost congruent trees with MP, ML and Bayesian methods. As in the separate analyses of ITS data set, *E. hainanensis* grouped with sect. *Crossadenia* from the New World with high support (PP = 1.00; BP = 100; Figure 3).

The metaphase chromosome morphology was shown in Appendix 3. *E. hainanensis* has a total chromosome number 2n = 4x = 28 (Table 1). Due to the insufficient seed materials, only six seeds germinated and the chromosomes photo were not suitable for further karyological analysis. The basic chromosome number x = 7 was estimatied according to published karyological studies.

**Table 1:** The chromosome structures for *Euphorbia*.

**Species**	**Subgenus/section**	**Chromosome number**	**Distribution**	**References**
*E. hainanensis*	subgen. *Chamaesyce* sect*. Hainanensis*	2n = 4x = 28	Hainan, China	This study
*E. acuta*	subgen. *Chamaesyce* sect. *Anisophyllum*	2n = 4x = 28 2n = 8x = 56	America continent	Urbatsch et al., 1975
*E. capitellata*	subgen. *Chamaesyce* sect. *Anisophyllum*	2n = 2x = 14	America continent	Urbatsch et al., 1975
*E. astyla*,	subgen. *Chamaesyce* sect. *Anisophyllum*	2n = 4x = 28	America continent	Urbatsch et al., 1975
*E. fendle*	subgen. *Chamaesyce* sect. *Anisophyllum*	2n = 8x = 56	America continent	Urbatsch et al., 1975
*E. maculata*	*subgen. Chamaesyce sect. Anisophyllum*	2n = 4x = 40 = 36m + 4sm, 2B	Beijing, China	Xue et al., 2007
*E. humifusa*	*subgen. Chamaesyce sect. Anisophyllum*	2n = 2x = 22 = 20m (2SAT) + 2sm, 2A	Beijing, China	Xue et al., 2007
*E. thymifolia*	subgen. *Chamaesyce* sect. *Anisophyllum*	2n = 4x = 40 = 40m, 2B	Beijing, China	Xue et al., 2007
*E. hirta*	subgen. *Chamaesyce* sect. *Anisophyllum*	2n = 2x = 18 = 14m + 4sm, 2B	Guangxi, China	Wang et al., 1999
*E. hypericifolia*	subgen. *Chamaesyce* sect. *Anisophyllum*	2n = 4x = 32 = 18m + 14sm, 2B	Guangxi, China	Wang et al., 1999
*E. angust*	subgen. *Chamaesyce* sect. *Anisophyllum*	2n = 2x = 14	America continent	Urbatsch et al., 1975
*E. lata*	subgen. *Chamaesyce* sect. *Anisophyllum*	2n = 4x = 28	America continent	Urbatsch et al., 1975
*E. polycarpa*	subgen. *Chamaesyce* sect. *Anisophyllum*	2n = 2x = 14	America continent	Urbatsch et al., 1975
*E. simulans*	subgen. *Chamaesyce* sect. *Anisophyllum*	2n = 2x = x14	America continent	Urbatsch et al., 1975
*E. theriaca*	subgen. *Chamaesyce* sect. *Anisophyllum*	2n = 2x = 14	America continent	Urbatsch et al., 1975
*E. heterophylla*	subgen. *Chamaesyce* sect *Poinsettia*	2n = 4x = 28	America continent	Aarestrup et al., 2008
*E. pulcherrima*	subgen. *Chamaesyce* sect *Poinsettia*	2n = 4x = 28 = 24m(3SAT) + 4sm, 2B	America continent	Xue et al., 2007
*E. eriantha*	subgen. *Chamaesyce* sect *Poinsettia*	2n = 4x = 28	America continent	Urbatsch et al., 1975
*E. cyathophora*	subgen. *Chamaesyce* sect *Poinsettia*	2n = 4n = 28 = 16m + 12sm, 2A	America continent	Xue et al., 2007
*E. exstipulata*	subgen. *Chamaesyce* sect *Poinsettia*	2n = 4x = 28	America continent	Urbatsch et al., 1975
*E. dentata*	subgen. *Chamaesyce* sect *Poinsettia*	2n = 4x = 28 = 28m, 1B	America continent	Xue et al., 2007
*E. marginata*	subgen. *Chamaesyce* sect. *Alectoroctonum*	2n = 8x = 56 = 40m + 8sm + 8st, 2B	America continent	Xue et al., 2007
*E. hylonoma*	subgen. Chamaesyce	2n = 2x = 20 = 16sm + 4st, 3A	Hubei, China	Wang et al., 1999
*E. helioscopia*	subgen. *Esula*	2n = 6x = 42 = 34m + 8sm, 1A	Hubei, China	Wang et al., 1999
*E. lathyris*	subgen. *Esula*	2n = 2x = 20 = 12m + 8sm, 2A	Sichuan, China	Xue et al., 2007
*E. esula*	subgen. *Esula*	2n = 2x = 20 = 14m + 6sm, 1A; 2n = 4n = 40 = 32m + 8sm, 1B	Beijing, China	Xue et al., 2007
*E. milii*	subgen. *Euphorbia*	2n = 4x = 40 = 32m + 8sm, 2B	America continent	Xue et al., 2007
*E. neriifolia*	subgen. *Euphorbia*	2n = 6x = 60 = 6m + 36sm + 12st + 6t, 3B	America continent	Xue et al., 2007
*E. antiquorum*	subgen. *Euphorbia*	2n = 6x = 60 = 24m + 24sm + 12st, 2B	India	Xue et al., 2007

## Discussion

In this study, biogeographic analysis with both ITS and cp DNA sequences revealed that *E. hainanensis* exhibited as a new section and showed no affinity with other *Euphorbia* species occurred in Hainan or the rest of the Old World, but closely related to subg. *Chamaesyce* sect. *Crossadenia* with high support, which are mainly distributed in South America of the New World. Additionally, we confirmed the results of several previous studies, which considered that *Euphorbia* subg. *Chamaesyce* is a strongly supported monophyletic group (Steinmann and Porter, 2002; Bruyns et al., 2006; Zimmermann et al., 2010; Horn et al., 2012; Yang et al., 2012). Both our ITS and combined ITS and cp DNA sequence phylogenetic analyses unambiguously grouped *E. hainanensis* with subg. *Chamaesyce* sect. *Crossadenia* (PP = 1.00, BP = 100; Figure 2). The karyological studies also suggests a close relationship with the New World *Euphorbia* subgen. *Chamaesyce* species, most of which has the basic chromosome number x = 7 and total chromosome number 2n = 14, 28 or 56 (Table 1; Urbatsch et al., 1975). However, the species of subgen. *Chamaesyce* occurring in the Eurasia usually have a different basic chromosome number (e.g., x = 8, 10 or 11; Table 1). Chromosome data for subgen. *Chamaesyce* sect. *Crossadenia* is presently unavailable and further inferences on the karyotype evolution of *E. hainanensis* and sect. *Crossadenia* are limited. The chromosomal structure for *Euphorbia* species, including the basic chromosome number and the polyploidy level, is still waiting to be studied precisely using genomic tools. The observation of morphological data approved our molecular and karyological data (Figure 1). Both *E. hainanensis* and all species of sect. *Crossadenia* shared common features: a terminal solitary cyathium, involucres unisexual or bisexual, styles 3 and basally connate, usually four light yellow glands, capsule well-exserted, subglobose to deeply 3-lobed and seeds globose (Figure 1). All this evidence suggests that the woody *E. hainanensis* was closely related to subg. *Chamaesyce* sect. *Crossadenia*. However, in the cp DNA sequence phylogenetic analyses, *E. hainanensis* did not form a monophyletic group with sect. *Crossadenia*, but branched at the base of sect. *Crossadenia*. The incongruity between nuclear ITS and cp DNA might be caused by complicated incomplete lineage sorting and/or introgression processes (Zhou et al., 2010, 2017). Further analyses showed that *E. hainanensis* divergent from section *Crossadenia* 9.3–30.6 Mya, and this period was considered as the most active period of plate movement.

It is well known that spatial or geographical barriers drive the origin of new species by fixing specific genotypes or morphological variations (Grant, 1981; Coyne, 1992; Gavrilets, 2003; Levin, 2003). Island-endemic species are assumed to originate from a widely-distributed species which originally distributed on the continental and then exposed to rapid geographical isolation events (Grant, 1981; Crawford and Smith, 1982; Crawford et al., 1985, 1993; Crawford, 2010). Most plant species in the western Pacific island chain, including those extending from Japan or Taiwan to the Philippines, possibly originated from temperate and tropical Asia, particularly from China (Huang et al., 2001; Hsieh, 2002; Chen, 2004; Chiang and Schaal, 2006; Takayama et al., 2013). For example, it is supposed that Taiwan-endemic spruce *Picea morrisonicola* derived from the ancestor *Picea wilsonii*, which widely occurred in the mainland base on population genetic evidence (Zou et al., 2013).

The Hainan Island is rich in endemic flora. A majority (505 species) of its recorded endemic plant species are originated from Eurasia continent or southeast Asia (Xing et al., 1995; Su et al., 2001). *Metapetrocosmea* and *Cathayanthe*, two endemic monotypic genera of *Gesneriaceae* in Hainan island, originated from the species that mainly distributed in Chinese mainland (Weber et al., 2011). However, *E. hainanensis* is restricted to the island of Hainan and isolated from related species by a long distance. The *Euphorbia* genus had experienced several long-distance dispersal events that led to its worldwide distribution and phylogenetic analyses showed sister species distributed in different continents (Yang et al., 2012; Peirson et al., 2013). As we known, the Hainan Island keeps a long distance from the America Island. So how did this dispersal happen? We hypothesize that birds may play an important role for the long-distance dispersal of *E. hainanensis* because we found that the seed coats become mucilaginous after 5 min in water. However, further population genetic investigations are still needed to verify our hypothesis and to clarify the speciation and migratory route.

## Taxonomic notes

Our results revised the taxonomic rank of *E. hainanensis* and considered its sole species to be a new section: *Euphorbia* subg. *Chamaesyce* sect. *Hainanensis*.

## Author contributions

XT conceived and designed the experiments. XT and QW performed the experiments and analyzed the data. XT contributed reagents, materials, analysis tools. XT and YZ wrote the paper.

### Conflict of interest statement

The authors declare that the research was conducted in the absence of any commercial or financial relationships that could be construed as a potential conflict of interest.
