# Supplementary material for: Euphorbia Section Hainanensis (Euphorbiaceae), a New Section Endemic to the Hainan Island of China From Biogeographical, Karyological, and Phenotypical Evidence
Source: Front Plant Sci. 2018 May 18;9:660. doi: 10.3389/fpls.2018.00660 (PMC5968112; doi:10.3389/fpls.2018.00660)

Supplementary material

Appendix A: Sequence information included in this study (nrDNA ITS, cpDNA *rbc*L and cp *ndhF*)

Species	Distribution/ vouchers	GenBank ITS	GenBank cp rbcL	GenBank cp ndhF
Euphorbia hainanensis	Exianling, Hainan, China, tianxm001	**MH**014759	**MH**014772 **MH**014773	**MH**014771
Euphorbia atoto	Changpo, Hainan, China, tianxm002	**MH**014762	**MH**014780	**MH**014768
Euphorbia hirta	Danzhou, Hainan, China, tianxm003	**MH**014758	**MH**014774	**MH**014765
Euphorbia thymifolia	Danzhou, Hainan, China, tianxm004	**MH**014757	**MH**014776	**MH**014766
Euphorbia heterophylla	Danzhou, Hainan, China, tianxm005	**MH**014761	**MH**014779	**MH**014769
Euphorbia_hyssopifolia	Lingshui, Hainan, China, tianxm006	**MH**014756	**MH**014777	**MH**014767
Euphorbia serrulata	Changjiang, Hainan, China, tianxm007	**MH**014760	**MH**014778	**MH**014764
Euphorbia cyathophora	Danzhou, Hainan, China, tianxm008	**MH**014763	**MH**014775	**MH**014770
Euphorbia tirucalli	Genebank	AF537479.1	JN249407.1	JN249234.1
Euphorbia neriifolia	Genebank	JN250199.1	JN249357.1	JN249187.1
Euphorbia humifusa	Genebank	KC758670.1	AB233884.1	JQ750817.1
Euphorbia milii	Genebank	AF537461.1	JN249349.1	AF538195.1
Euphorbia maculate	Genebank	JQ750939.1	JN249345.1	JQ750829.1
Euphorbia cotinifolia	Genebank	JQ750901.1	JN249286.1	JQ750783.1
Euphorbia pulcherrima	Genebank	AF537432.1	JN249377.1	AF538168.1
Euphorbia cyathophora	Genebank	GU214931.1	JQ750904.1	JQ750786.1
Euphorbia dentate	Genebank	KC758668.1	JN249292.1	JQ750789.1
Euphorbia lathyris	Genebank	AF537550.1	JN249338.1	JN249170.1
Euphorbia helioscopia	Genebank	JQ750880.1	JN249326.1	JQ750809.1
Euphorbia peplus	Genebank	AF537532.1	JN249367.1	JN249197.1
Euphorbia radians	Genebank	AF537438.1	JN249380.1	AF538169.1
Pedilanthus tithymaloides	Genebank	AF537494.1	AB267959.1	AF538216.1
Euphorbia eriantha	Genebank	AF537440.1	JN249300.1	AF538167.1
Euphorbia graminea	Genebank	HQ645263.1	JN249318.1	AF538155.1
Euphorbia appariciana	Genebank	AF537455.1	JN249270.1	AF538177.1
Euphorbia hadramautica	Genebank	JN250166.1	JN249324.1	JN249156.1
Euphorbia glanduligera	Genebank	AF537426.1	JN249315.1	AF538178.1
Euphorbia cornastra	Genebank	JQ750899.1	JN249285.1	JQ750781.1
Euphorbia didiereoides	Genebank	JQ952319.1	JQ952171.1	JQ952171.1
Euphorbia marginata	Genebank	AJ534788.1		

Euphorbia stracheyi	Genebank	KF264461.1	KC212647.1
Euphorbia rapulum	Genebank	KC212358.1	KC212616.1
Euphorbia alatavica	Genebank	GU953741.1	
Euphorbia pilosa	Genebank	AF537557.1	AF538234.1
Euphorbia officinarum	Genebank	JN207762.1	
Euphorbia altaica	Genebank	GU979429.1	
Euphorbia micractina	Genebank	KC212309.1	KC212571.1
Euphorbia lucorum	Genebank	EU659771.1	
Euphorbia pekinensis	Genebank	GU441821.1	
Euphorbia jolkinii	Genebank	KC212284.1	KC212548.1
Euphorbia peplus	Genebank	AF537532.1	
Euphorbia franchetii	Genebank	KC212256.1	KC212516.1
Euphorbia inderiensis	Genebank	KC212277.1	
Euphorbia dracunculoides	Genebank	HQ830198.1	KC212494.1
Euphorbia latifolia	Genebank	GU984316.1	
Euphorbia sieboldiana	Genebank	EU659753.1	
Euphorbia esula	Genebank	AF537546.1	
Euphorbia oaxacana	Genebank	AF537373.1	
Euphorbia guatemalensis	Genebank	AF537408.1	
Euphorbia rzedowskii	Genebank	AF537399.1	
Euphorbia delicatula	Genebank	AF537393.1	AF538152.1
Euphorbia ceroderma	Genebank	AF537389.1	AF538153.1
Euphorbia succedanea	Genebank	AF537403.1	AF538162.1
Euphorbia misella	Genebank	AF537384.1	AF538160.1
Euphorbia articulata	Genebank	AF537446.1	AF538175.1
Euphorbia bilobata	Genebank	AF537435.1	AF538172.1
Euphorbia mertonii	Genebank	HQ645351.1	
Euphorbia cumbrae	Genebank	HQ645252.1	
Euphorbia zambesiana	Genebank	HQ645368.1	
Euphorbia chamaerrhodos	Genebank	HQ645245.1	
Euphorbia umbellulata	Genebank	HQ645363.1	
Euphorbia remyi	Genebank	HQ645331.1	
Euphorbia simulans	Genebank	HQ645347.1	
Euphorbia acuta	Genebank	AF537450.1	AF538176.1
Euphorbia johnstonii	Genebank	HQ645287.1	
Euphorbia angusta	Genebank	HQ645222.1	JQ750763.1
Euphorbia planiticola	Genebank	KC019498.1	JQ750839.1
Euphorbia guiengola	Genebank	JQ750919.1	JQ750805.1
Euphorbia gueinzii	Genebank	JQ750917.1	JQ750803.1
Euphorbia phylloclada	Genebank	AF537427.1	AF538179.1
Euphorbia sarcodes	Genebank	AF537454.1	JQ750847.1
Euphorbia goyazensis	Genebank	JQ750914.1	JQ750799.1
Euphorbia lycioides	Genebank	JQ750936.1	JQ750826.1
Euphorbia crossadenia	Genebank	AF537451.1	

Euphorbia petiolata	Genebank	AF537422.1	
Euphorbia cheirolepis	Genebank	AF537424.1	
Euphorbia sphaerorhiza	Genebank	AF537412.1	AF538158.1
Euphorbia macropodoides	Genebank	JQ750937.1	JQ750827.1
Euphorbia wallichii	Genebank	KC212426.1	KC212683.1
Euphorbia turczaninowi	Genebank	AF537543.1	KC212668.1

Appendix B Primers used for amplification and sequencing in this study.

Primer name	sequence	Primer origin
*rbc*L-1FS	ATGTCACCACAAACAGAAAC	Fay et al., 1997
*rbc*L-R	TCACAAGCAGCTAGTTCAGGACTC	Pryer et al.,2001
ITS-1F	GTCCACTGAACCTTATCATTTAG	White et al., 1990
ITS-R	TCCTTCCGCTTATTGATATGC	White et al.,1990
*ndh*F536F	TTGTAACTAATCGTGTAGGGGA	Steinmann and Porter, 2002
ndhF1318R	CGAAACATATAAAATGCRGTTAATCC	Steinmann and Porter, 2002
ndhF972F	GTC TCA ATT GGG TTA TAT GAT G	Steinmann and Porter, 2002
*ndh*F*2110Ri*	TCAATTATTCGTTTATCAA	Steinmann and Porter, 2002

Appendix 1 Bayes tree based on *ndh*F data set. Numbers after brahches are Bayes

posterior probabilities. The branches of *E. hainanensis* were marked in red.

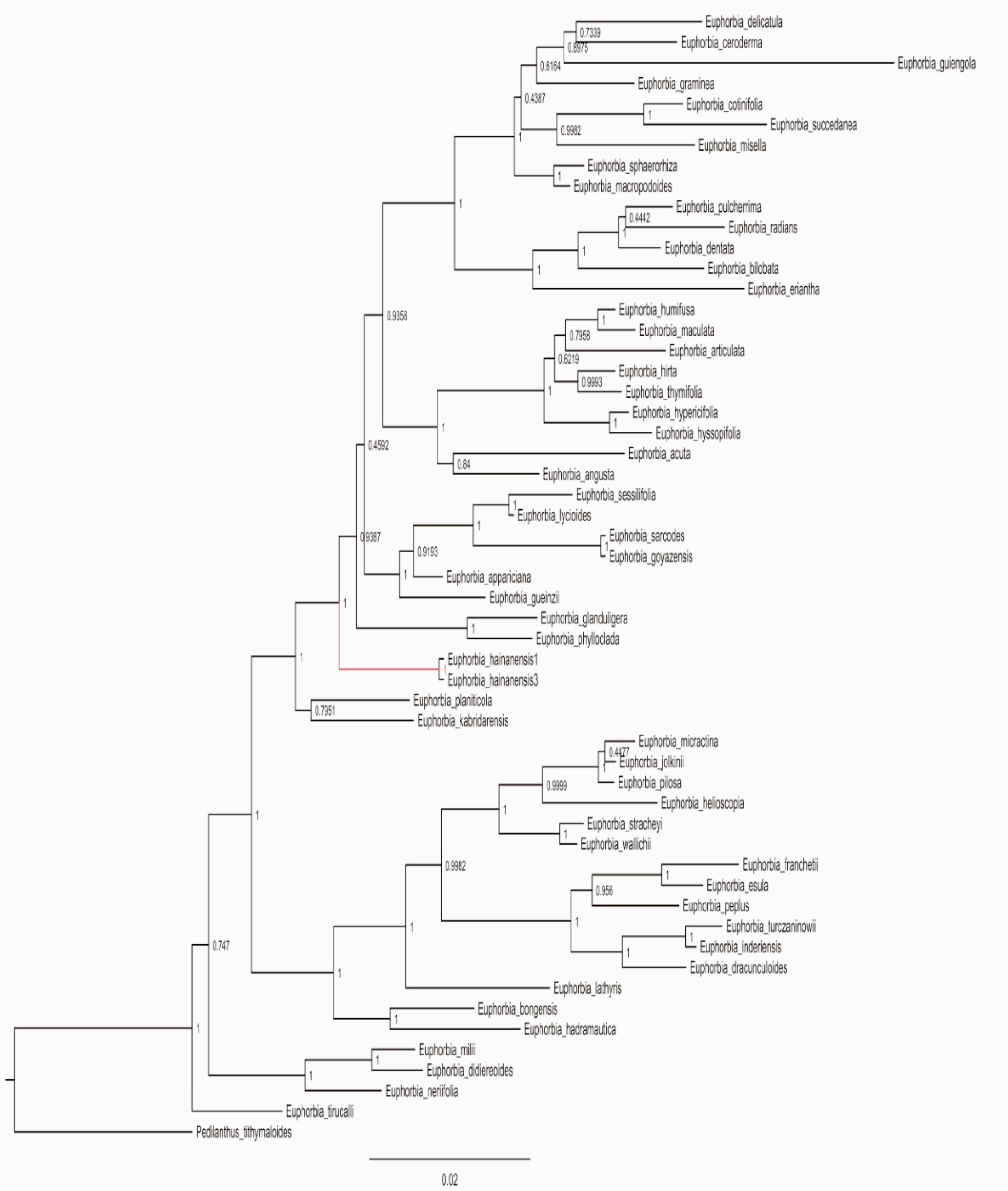

Appendix 2 Maximum Likelihood tree based on analysis of the concatenated cpDNA data set. Numbers on branches are Bayes posterior probabilities/MP bootstrap values. Values < 50% are not shown. Branches leading to *E. hainanensis* are shown in red.

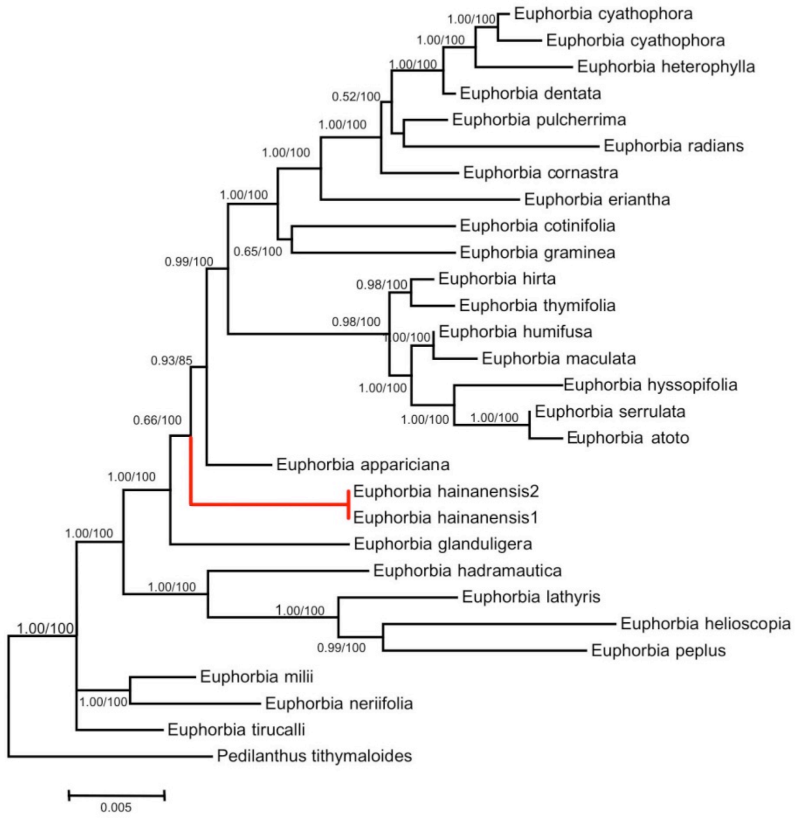

Appendix 3 Chromosomes at metaphases for *E. hainanensis*.

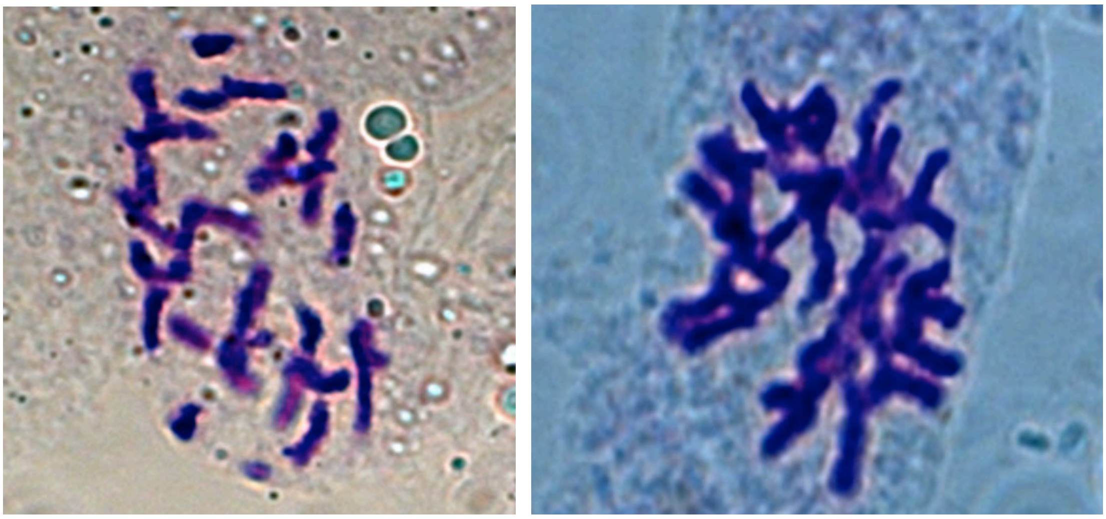

Supplement: Supplementary file 1 [file Data_Sheet_1.pdf]
